# Budget impact analysis of temocillin insurance coverage for urinary tract infections caused by ESBL-producing pathogens in Iran

**DOI:** 10.1097/MD.0000000000034436

**Published:** 2023-09-01

**Authors:** Meysam Seyedifar, Menhajuddin Sabouri, Omid Soodi, Hananeh Ghasemi

**Affiliations:** a Pharmaceutical Management and Economic Research Center, The Institute of Pharmaceutical Sciences (TIPS), Tehran University of Medical Sciences, Tehran, Iran; b Faculty of Pharmacy, Tehran University of Medical Sciences, Tehran, Iran.

**Keywords:** antimicrobial resistance, antimicrobial stewardship, carbapenem-sparing strategy, cost, economic evaluation, temocillin, third-party payer, urinary tract infection

## Abstract

**Background:**

One of the most prevalent infections with a significant disease burden is urinary tract infections (UTIs), which occurs in approximately 50% of women at least once in their lifetime. Antimicrobial resistance to pathogens causing UTIs is expanding worldwide and has been associated with increased use of broad-spectrum antibiotics, including carbapenems, leading to significant costs for insurance and healthcare systems. The emergence of resistance to carbapenems has led to an increasing need for and interest in carbapenem-sparing strategies, including the use of narrow-spectrum antibiotics, such as temocillin. Temocillin has a strong bactericidal effect, along with high tolerability and a good safety profile. It is also stable toward most extended-spectrum beta-lactamases (ESBL). The purpose of our study was to design a budget impact analysis (BIA) model and estimate the budget impact of temocillin insurance coverage for the treatment of UTIs caused by ESBL-producing bacteria from the perspective of the payer.

**Methods:**

The BIA model with insurance payer perspective was used to estimate the impact of temocillin insurance coverage on the treatment of UTIs caused by ESBL-producing bacteria over a 1-year time horizon in Iran. Direct medicine costs, hospitalization and paraclinical costs, and side effect costs were considered in this model. To assess the impact of uncertainty on the model inputs, 1-way sensitivity analyses were performed.

**Results:**

The model demonstrates that inclusion of temocillin in insurance coverage, decreasing treatment costs from $36 million to $34 million, will result in overall savings of > $1.9 million and lead to > $9 million savings in insurance costs for antimicrobial resistance.

**Conclusion:**

The inclusion of temocillin in the insurance coverage in Iran for patients developing UTIs caused by ESBL-producing bacteria would be cost-saving for insurance and decrease the risk associated with emerging antimicrobial resistance.

## 1. Introduction

One of the most common infections causing a significant disease burden is a urinary tract infection (UTI). The global prevalence of UTI is estimated to be 150 million patients annually.^[1]^ According to a report by the World Health Organization, approximately 50% of women experience UTI at least once in their lifetime. According to this report, UTIs account for 8.3 million office visits and more than 1 million hospitalizations annually.^[2]^

The antimicrobial resistance of pathogens causing UTIs is expanding worldwide. The decreased susceptibility of these microorganisms to common antibiotics has changed the pattern of antibiotic prescription and expanded the use of broad-spectrum antibiotics, including carbapenems. This can increase the risk of developing antimicrobial resistance in the long term. Additionally, the emergence of carbapenemase enzymes has led to increased resistance to carbapenems.^[3]^ Healthcare systems in Europe and Asia have encountered growing concerns with extended-spectrum beta-lactamases (ESBL)-producing *Escherichia coli*, especially CTX-M-producing strains, particularly among patients with complex conditions and those exposed to antibiotic therapy and hospitalization.^[4]^ In a study by Kashef et al, evaluating the geographical pattern of isolated resistant pathogens from UTI cases in Tehran, *E coli* was found to be the most commonly isolated microorganism (68.8%), followed by *Proteus* spp. (12.4%) and *Klebsiella* spp. (9.6%).^[5]^ Therefore, narrow-spectrum antibiotics with high efficacy in UTIs can be a suitable alternative to broad-spectrum antibiotics such as carbapenems.

Temocillin, a 6-α-methoxy derivative of ticarcillin, is a bactericidal antibiotic belonging to the class of beta-lactams.^[6]^ Temocillin has a strong bactericidal effect on *E coli, Proteus* spp., *Klebsiella* spp., *Citrobacter* spp., other Enterobacterales, and to a lesser extent, *Serratia* species.^[7,8]^ Temocillin is stable against most classic ESBL, including TEM, SHV, CTX-M, and AmpC beta-lactamases.^[9,10]^ Moreover, temocillin has a high tolerability and a good safety profile. However, this is contraindicated in cases of hypersensitivity to penicillin.^[11]^ Due to its relatively narrow spectrum of activity and stability towards ESBL, temocillin is a suitable option for targeted treatment based on microbiological diagnosis, especially in UTIs caused by ESBL-producing bacteria. Temocillin can therefore be an alternative to carbapenems, which are considered as the reference treatment for such resistant bacteria.^[12,13]^

In a study by Nguyen et al, the cost-effectiveness of temocillin *versus* meropenem was demonstrated in the treatment of UTIs caused by ESBL-producing bacteria, using a combined decision analytic and Markov model over a 5-year period.^[14]^ In addition to cost-effectiveness analysis (CEA), budget impact analysis (BIA) is an important part of health economic studies, which estimates the financial consequences of the adoption of new therapeutic interventions. BIA models predict the financial impact and cost savings resulting from new policies or programs in the healthcare system, and estimate the impact of cost savings on patient access to treatment.^[15]^

The purpose of our study was to design a BIA model and estimate the budget impact of temocillin insurance coverage in the treatment of UTIs caused by ESBL-producing bacteria from the perspective of the payer.

## 2. Materials and Methods

### 2.1. Model overview and time horizon

This BIA model with insurance payer perspective estimated the impact of insurance coverage of temocillin for UTIs caused by ESBL-producing bacteria over a 1-year period in Iran. Two scenarios were hypothesized in the model: in the first scenario, treatment costs for UTIs caused by ESBL-producing bacteria were calculated based on the current situation under which temocillin is not covered by insurance, and in the second scenario, costs were estimated assuming temocillin insurance coverage.

### 2.2. Defining target population

To estimate the population size of the model, the overall prevalence of UTIs was determined to be 11% of the total population in Iran, similar to the prevalence of UTIs reported in the United States.^[16]^ A study by Foster et al was used to estimate the population with complex (c)UTIs caused by ESBL-producing bacteria. In this study, out of 32,521,146 patients who developed UTI, only 297,470 (0.91%) were hospitalized.^[17]^ For greater accuracy, among hospitalized patients with UTIs, it was estimated that 66.7% of infections in inpatients were caused by ESBL-producing bacteria, according to a study by Ranjan et al.^[18]^ Therefore, the target population was estimated to be 53,689 individuals.

### 2.3. Determining costs and probabilities

Costs were calculated from an insurance perspective. For this purpose, government tariffs and prices approved by insurance were considered for calculation, and according to the coverage of 90% of insurance in hospitalized patients, this amount of coverage was calculated and applied for costs. Costs were divided into 3 categories: direct medicine costs, inpatient and paraclinical costs (including bed days, nursing, visits, counseling, tests, and imaging), and side effects costs. A 2-level decision tree model was designed for first-level patients receiving first-line treatment, according to each antibiotic prescription share; based on the cure rate of each antibiotic, some patients were not cured and received second-line therapy, which is assumed to be one of the other available options.

#### 2.3.1. Direct drug cost.

Medicine costs were calculated and based on the approved price of each drug, treatment regimen, dose used in the treatment of the study indication, and the estimated length of hospital stay. Direct medication costs were calculated based on approved prices available on the Iran Food and Drug Administration (FDA) website and the Social Security Organization website. In addition, the therapeutic dose of the medications was determined based on the dose recommendations mentioned in the guidelines and medication labels. The coverage of all medications used in the hospital was 90%, and that of ciprofloxacin was 70%. Levofloxacin did not have insurance coverage; therefore, its direct cost was not considered in this calculation. In the second scenario, the temocillin insurance coverage was assumed to be 90%, similar to that of other hospitalized drugs. Details of the antibiotic treatment costs are presented in Table 1. Treatment duration was estimated based on the perspective of the clinician and usual practice in Iran and adjusted by FDA label, clinical studies, and real-world data for levofloxacin and temocillin.^[19]^ There are different available data on temocillin treatment duration, and for better comparability with the perspective of the clinician, real-world data were used.

**Table 1:** Costs of antibiotic therapy (USD).

Antibiotic (dosage)	Fee price ($)	Daily consumption*(g)	Treatment duration (d)	Total cost ($)
Imipenem + cilastatin (500 + 500 mg)	5.76	7	12	484
Meropenem (1 g)	6.43	3	12	231.43
Piperacillin/Tazobactam (3.375 g)	2.98	4	12	142.86
Levofloxacin (750 mg)	0.75	1	5	3.75
Ciprofloxacin (500 mg)	0.14	2	12	3.29
Temocillin (1 g)	27.62	4	6	662.86

* Local expert panel.

#### 2.3.2. Inpatient and paraclinical cost.

Hospitalization and paraclinical insurance costs were calculated and based on governmental fees for medical services (Table 2). According to internal specialists, the length of hospitalization in the ward and intensive care unit (ICU) was 12 and 4 days, respectively. The hospitalization rates in the ICU and ward were 3% and 97%, respectively, based on the study by Parienti et al.^[20]^

**Table 2:** Hospitalization and paraclinical costs.

Parameters	Cost (USD)	
	Medical ward	ICU
Bed-day (12 d in ward and 4 d in ICU)	353.71	1265.52
6% nursing	21.22	75.93
Day 1 visit	14.59	14.59
Visit after d 2	131.29	155.16
Discharge visit	9.28	9.28
Counseling	43.76	43.76
Tests	38.29	68.85
Imaging	26.57	26.57
Total hospitalization and paraclinical costs in ward	638.72	
Total hospitalization and paraclinical costs in ward and ICU		1659.68

ICU = intensive care unit.

#### 2.3.3. Side effects cost.

The most important and costly side effect of antibiotics is *Clostridioides difficile*-associated diarrhea. Temocillin is reported to be a low inducer of diarrhea and *C difficile* infection (CDI).^[21]^ Considering this, only the cost of diarrhea caused by *C difficile* was considered, and other costs were excluded. According to the results of a meta-analysis by Marra et al, the baseline incidence of CDI is 8.3 per 10,000 people.^[22]^ According to the results of a study by Teng et al, the incidence of CDI was associated with the use of various antibiotic classes, and the relationship was calculated and reported as an odds ratio (OR).^[23]^ To convert the OR to Risk Ratio (RR), the following formula was used, where P_ref_ is the prevalence of the outcome in the reference group:


$$\begin{array}{l} RR=OR/\left(\left(1-P_{ref}\right)+\left(P_{ref}*OR\right)\right)\; \end{array}$$

To calculate CDI costs, owing to the high share of hospitalization costs, only the increased hospitalization costs were calculated. According to a meta-analysis by Zhang et al, 9.7 days was used as the increased length of hospitalization associated with CDI.^[24]^ By multiplying the baseline probability of CDI occurrence in RR, the length of hospital stay, the cost of each hospital bed, and the insurance coverage (90%), the cost of CDI per patient was calculated for different antibiotic treatments (Table 3). The OR of temocillin is assumed to be the same as that of average antibiotics (OR = 1), although Livermore et al suggested little or no propensity of temocillin to select for overgrowth by *C difficile*.^[6]^

**Table 3:** Rates and costs of Clostridioides difficile infections per antibiotic agent.

Antibiotic agent	OR	RR	CDI rate	CDI cost per patient ($)
Imipenem	5.77	5.68	0.0047144	1.35
Meropenem	26.58	24.51	0.0203433	5.82
Piperacillin/Tazobactam	30.58	35.11	0.0291413	8.33
Levofloxacin	1.93	1.92	0.0015936	0.46
Ciprofloxacin	8.03	7.8	0.006474	1.85
Temocillin	1	1	0.00083	0.24

CDI = Clostridioides difficile infection, OR = odds ratio, RR = risk ratio.

#### 2.3.4. Antimicrobial resistance cost.

Because of the significant cost of antimicrobial resistance, these costs were explored and calculated in our study to estimate the impact of temocillin insurance coverage. UTIs account for a large percentage of all infections, and there is a high prevalence of antimicrobial resistance to carbapenems, as well as a correlation between carbapenem use and the incidence of carbapenem resistance. Therefore, a direct and linear relationship between carbapenem use and antimicrobial resistance was assumed (correlation coefficient of 80%). Due to the lack of national reports, existing studies on the cost of antimicrobial resistance and its ratio to the total health expenditure in the United States were used to estimate the cost of antimicrobial resistance.

The cost of antimicrobial resistance and total healthcare spending in the United States were estimated at $55 billion and $2.9 trillion, respectively, in 2013.^[25]^ Thus, the ratio of antimicrobial resistance treatment costs to the total healthcare spending in the United States was 0.69%, which was assumed to be the same for Iran. Based on expert opinions, total healthcare insurance spending was estimated at approximately $13.1 billion, which was used for our model. Based on the calculated ratio, antimicrobial resistance costs more than $90 million for healthcare insurance.

To calculate the decline in antimicrobial resistance cost due to the decreasing use of broad-spectrum antibiotics, the reduction in carbapenem use was calculated in the second scenario (after temocillin insurance coverage). Accordingly, following temocillin insurance coverage, a 30% increase in its use for cUTIs is expected, with equivalent reduction in the share of other antibiotics, and a decrease in carbapenem use by 17% is hypothesized. According to the statistics of the Iran FDA, this leads to an average decline of 12% in the use of imipenem and meropenem.

Based on previous assumptions, from the product of 12% multiplied by 80% (correlation coefficient) and $90 million, the decrease in insurance costs is estimated at > $9 million following the reduction in broad-spectrum antibiotic use and antimicrobial resistance resulting from temocillin insurance coverage.

### 2.4. Antibiotic cure rate estimation

For our budget impact model, we required the estimated cure rate of each common antibiotic in the treatment of upper UTI caused by ESBL-producing bacteria. To estimate the current prescription rate of different antibiotics, a questionnaire was designed to collect responses from 19 infectious disease specialists (Table 4). The cure rates of antibiotics were assessed through a review of existing literature (Table 5). Because of variations in the response to different antibiotics by time and place, together with inconsistent cure rates due to antimicrobial resistance, we attempted to estimate the cure rates of antibiotics based on up-to-date studies, which is appropriate for circumstances in Iran. Table 5 depicts the cure rates of antibiotics in the treatment of complex pyelonephritis caused by ESBL-producing bacteria.

**Table 4:** Estimated prescription rates of antibiotics for the treatment of complicated pyelonephritis prior to and post-coverage of temocillin by expert panel.

Antibiotic agent	Prior to coverage (current)	Post-coverage
Imipenem	27.50%	19.00%
Meropenem	27.00%	18.50%
Piperacillin/Tazobactam	17.50%	12.00%
Levofloxacin	10.00%	6.50%
Ciprofloxacin	9.50%	6.00%
Temocillin	8.50%	38.00%

**Table 5:** Cure rates of antibiotics in the treatment of complicated pyelonephritis caused by ESBL-producing bacteria.

Antibiotic agent	Cure rate	Reference
Imipenem	82%	(Edwards et al, 2005)^[26]^
Meropenem	85%	(Nguyen et al, 2019)^[14]^
Piperacillin/Tazobactam	88%	(Nguyen et al, 2019)^[14]^
Fosfomycin	71%	(Bielen & Likic, 2019)^[27]^
Levofloxacin	72%	(Reza Mortazavi-Tabatabaei et al, 2019)^[28]^
Ciprofloxacin	72%	(Reza Mortazavi-Tabatabaei et al, 2019)^[28]^
Temocillin	93%	(Nguyen et al, 2019)^[14]^

ESBL = extended-spectrum beta-lactamases.

### 2.5. Assumptions in the model

Due to the absence of related literature and statistics on Iran, we assumed that the ratio of UTI prevalence to the total population and the ratio of antimicrobial resistance cost to total healthcare costs for Iran were equal to those in the United States. However, this assumption does not have a negative impact on the results because of the unfavorable situation in Iran regarding antimicrobial resistance, which suggests higher antimicrobial resistance costs in Iran than in the US. In the calculation of side effects cost, we only considered the costs of diarrhea caused by *C difficile* and excluded other side effects. This assumption strengthens our results, as, based on reviewed literature, temocillin may also have fewer side effects than other antibiotics.

### 2.6. Sensitivity analysis

To assess the impact of uncertainty on model inputs, 1-way sensitivity analyses were carried out by varying the parameters of the model, such as hospitalization days, temocillin price, imipenem treatment duration, the same cure rate for all antibiotics, and CDI length of hospital stay, within reported upper and lower limits or by ± 20% for parameters without upper and lower limits. In addition, the correlation between carbapenem usage and antimicrobial resistance was considered to have a coefficient of 80% and varied by ± 10%.

## 3. Results

### 3.1. BIA

Based on the assumptions and inputs, we conducted a BIA under a base-case scenario (without insurance coverage of temocillin) and the temocillin coverage scenario. The details are presented in Tables 6 and 7. As cUTI is an acute condition, the budget impact was calculated for a 1-year period. The results are presented in Table 8.

**Table 6:** Costs of treatment with different antibiotics in the first scenario (prior to temocillin coverage).

Antibiotic agent	Number of patients in first-line therapy	Number of patients in second-line therapy	Cost (USD)
Imipenem	14764	2697	14,367,878.37
Meropenem	14496	2174	11,569,471.35
Piperacillin/Tazobactam	9396	1127	6899,473.04
Levofloxacin	5369	1503	874,923.07
Ciprofloxacin	5100	1428	798,857.83
Temocillin	4564	319	1512,963.50
Total cost			36,023,567.17

**Table 7:** Costs of treatment with different antibiotics in the second scenario (post-temocillin coverage).

Antibiotic agent	Number of patients in first-line therapy	Number of patients in second-line therapy	Cost (USD)
Imipenem	10201	1864	10,065,913.61
Meropenem	9932	1490	7992,464.04
Piperacillin/Tazobactam	6443	773	4756,701.03
Levofloxacin	3490	977	685,286.88
Ciprofloxacin	3221	902	640,850.75
Temocillin	20402	1428	18,509,766.64
Total cost			34,083,183.28

**Table 8:** Total costs and budget impact in both scenarios (USD).

Total costs in the first scenario (prior to temocillin coverage)	36,023,567.2
Total costs in the second scenario (post-temocillin coverage)	34,083,183.3
Budget impact of temocillin coverage	−1940,383.9

The BIA demonstrates that inclusion of temocillin in insurance coverage would result in cost savings of > $1.9 million, decreasing treatment costs from $36 million to $34 million.

### 3.2. Sensitivity analysis

**Figure 1.**
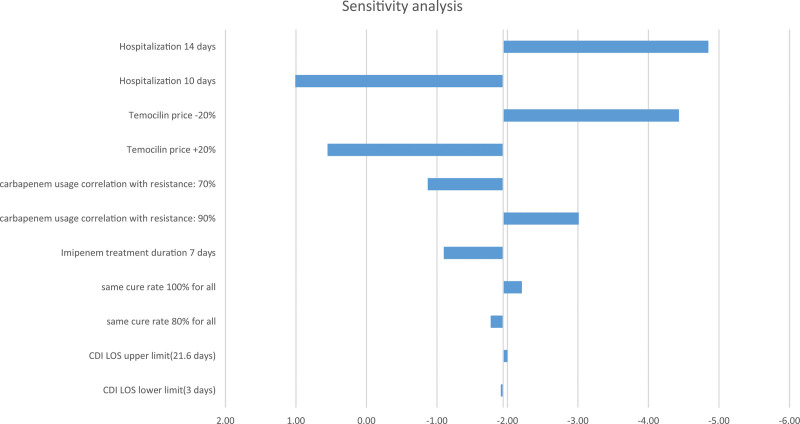
Tornado diagram of sensitivity analysis.

To measure the precision and strength of the results, their sensitivity to various inputs and assumptions, and to explore the impact of the 11 variables on the model, a sensitivity analysis was conducted. As the tornado diagram illustrates (Fig. 1), the model is highly sensitive to decreasing the hospitalization length to 10 days and increasing the price of temocillin by 20%. Other inputs have a relatively slight impact on the results, which suggests that the model remains stable, and the coverage scenario is still cost saving.

## 4. Discussion

UTIs, one of the most prevalent infections, particularly among women, carry a significant disease burden. According to a World Health Organization report, UTIs account for 150 million cases of infection, 8.3 million office visits, and more than 1 million hospitalizations annually.^[2]^ Moreover, vulnerable populations, including infants, pregnant women, the elderly, diabetic or multiple sclerosis patients, and immunocompromised or catheterized patients are more likely to be affected by UTI. Catheter-associated UTI are also responsible for most nosocomial infections.^[29]^

Antimicrobial resistance is a critical issue in healthcare systems and leads to significant direct and indirect societal costs.^[30,31]^ Antibiotic stewardship programs were implemented to cut off and control these costs. Overuse of broad-spectrum antibiotics, including carbapenems, leads to antimicrobial resistance to these agents, particularly in bacterial species and opportunistic bacteria, such as *Pseudomonas* and *Acinetobacter*. Sparing broad-spectrum antibiotics using narrow-spectrum antibiotics is crucial in antibiotic stewardship to control antimicrobial resistance.^[32]^ A previous study on prediction of the impact of the antibiotic stewardship policy showed that the decline in meropenem use resulted in a decrease in the emergence of OXA-48 enzyme-producing resistant *Klebsiella* strains.^[33]^ Another study conducted 1999 to 2001 in the US found a strong correlation between meropenem use (in defined daily dose per patient bed) and *Pseudomonas* resistance (*R* = 0.98).^[34]^ Sistani et al demonstrated that a 60% decrease in imipenem use significantly increased the sensitivity of isolated *Pseudomonas* strains in a hospital ICU in Tehran.^[35]^ We studied the literature on the rates of resistance to imipenem, meropenem, and colistin in Iran. These results point to the growing concern regarding antimicrobial resistance, particularly to colistin, which is one of the most effective agents against nosocomial infections, to which resistance may lead to higher mortality and morbidity.

Temocillin, as a narrow-spectrum antibiotic, can be a promising carbapenem-sparing agent for the treatment of UTI with a high bactericidal effect on pathogens such as *E coli* and other Enterobacterales and, simultaneously, a high tolerability and acceptable side effects profile.^[8,11]^ This study was conducted from the perspective of insurance payers to evaluate the budget impact of temocillin inclusion in insurance coverage on saving costs related to UTIs treatment and antimicrobial resistance. The findings of the analyses suggest that the second scenario (temocillin coverage) can result in savings of more than $1.9 million and leads to more than $9 million saving in the insurance costs for antimicrobial resistance. To evaluate the uncertainty of the model inputs, sensitivity analyses were conducted. The results showed that the model is robust, and among different parameters, decreasing hospitalization length to 10 days and increasing the price of temocillin by 20% influenced the model.

However, our study has some limitations, and there are simplifications to our assumptions. Given the lack of studies and reports related to the antimicrobial resistance ratio or UTI and hospitalization prevalence in Iran, we used reported values for the US. To estimate the cost of side effects, we only considered the costs of *C difficile* diarrhea and excluded other side effects.

## 5. Conclusions

Based on the findings of our study and considering the advantages of narrow-spectrum antibiotics over broad-spectrum antibiotics in lowering the risk of antimicrobial resistance, the inclusion of temocillin in the insurance coverage for patients developing UTIs caused by ESBL-producing bacteria may lead to improved antibiotic stewardship and consequently save costs by more than $1.9 million. Thus, temocillin coverage would be cost saving and would not impose a significant cost burden on insurance.

## Author contributions

**Conceptualization:** Meysam Seyedifar, Menhajuddin Sabouri.

**Data curation:** Meysam Seyedifar, Hananeh Ghasemi.

**Formal analysis:** Meysam Seyedifar.

**Investigation:** Meysam Seyedifar.

**Methodology:** Meysam Seyedifar, Omid Soodi.

**Supervision:** Meysam Seyedifar.

**Writing – original draft:** Menhajuddin Sabouri, Omid Soodi.

**Writing – review & editing:** Meysam Seyedifar, Menhajuddin Sabouri.
